# Personal radio use and risk of cancers among police officers in Great Britain: Results from the airwave health monitoring study

**DOI:** 10.1002/ijc.70255

**Published:** 2025-11-18

**Authors:** Chiara Di Gravio, Paul Elliott, David C. Muller

**Affiliations:** ^1^ MRC Centre for Environment and Health, Department of Epidemiology and Biostatistics, School of Public Health Imperial College London UK; ^2^ National Institute for Health and Care Research Health Protection Research Unit in Radiation Threats and Hazards, Department of Epidemiology and Biostatistics, School of Public Health Imperial College London UK

**Keywords:** cancer, epidemiology, occupational health, radio‐frequency exposure

## Abstract

Exposure to radiofrequency electromagnetic fields (RF‐EMF) from mobile phones and other wireless devices has been classified as possibly carcinogenic to humans. With data from 48,457 police officers and staff enrolled in the Airwave Health Monitoring Study, we investigated associations between personal radio use and the risk of developing cancer using Cox proportional hazard regressions. Personal radio use and duration of use were derived by combining objective data on call duration provided by the Home Office and participants' self‐reported data via gradient boosting methods. Across a median follow‐up time of 11 years, there were 1502 incident cancer cases of which 146 were cancers of the head, neck and central nervous system (CNS). There was no association between personal radio use, all cancers (hazard ratio [HR] = 0.96, 95% confidence interval [CI]: 0.79, 1.15) and head, neck, and CNS cancers (HR = 0.74, 95% CI: 0.39, 1.38). Doubling minutes of call duration via personal radio use was not associated with increased hazard of developing all cancers (HR = 1.00, 95% CI: 0.96, 1.04) or head, neck and CNS cancers (HR = 1.09, 95% CI: 0.97, 1.22). Results were similar when considering exposure to RF‐EMF via mobile phone use as well as when restricting the analyses to police officers only.

## Graphical abstract

What's new?

Terrestrial trunked radio (TETRA) is the standard digital communication system used by police and other emergency services in Great Britain. During active use, TETRA emits radiofrequency electromagnetic fields, which are potentially carcinogenic in humans. Here, using data on objective call duration, the authors examined cancer risk from personal radio use among police forces across Great Britain. No significant association was detected between radio use, call duration, and cancer risk.

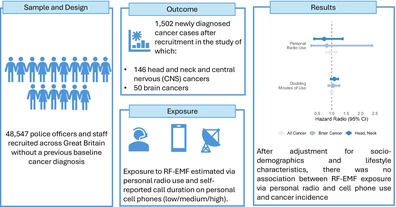

**Abbreviations**
BMI: body mass index
CI: confidence interval
CNS: central nervous system
GSM: global system for mobile communication
HR: hazard ratio
IQR: interquartile range
MN: malignant neoplasm
PSTN: public switch telephone network
PTT: press‐to‐talk
RF‐EMF: radiofrequency electromagnetic fields
SAR: specific energy absorption rate
TETRA: terrestrial trunk radio

## 1 INTRODUCTION

Understanding the potential long‐term impact of mobile phones and other wireless technology on health is of high importance, given their widespread use. There have been multiple studies linking exposures to radiofrequency electromagnetic fields (RF‐EMF) and health outcomes with a focus on cancer,^1^, ^2^, ^3^ heart rate variability^4^, ^5^, ^6^ and cognitive function.^7^, ^8^ Based on studies of mobile phone use and brain cancer, the International Agency for Research on Cancer (IARC) classified exposure to RF‐EMF as possibly carcinogenic to humans, with limited and inconsistent evidence of carcinogenicity.^9^

Police forces and other emergency services across Great Britain have used terrestrial trunked radio (TETRA) as a digital communication system since 2001. Specific energy absorption rate (SAR) in the head for a typical TETRA handset varies from 1.3 to 4.0 W kg^−1^ suggesting that exposure—while being below occupational guidelines—could exceed general population guidelines.^10^ TETRA differs from GSM (global system for mobile communication) mobile phone technology in terms of average output power and transmission frequency with mobile phone transmission pulsed at 217 Hz, and TETRA signal pulsed at 17.6 Hz. Moreover, as RF‐EMF is emitted by TETRA only when actively talking on the radio and not when in listening mode, mobile phone and TETRA use may have different effects on long‐term health outcomes.

To address any concern related to the use of TETRA among British police officers, in 2004 the Home Office commissioned the Airwave Health Monitoring. In previous analyses in Airwave, TETRA use was not associated with the risk of cancer,^1^ but the small number of cancers, especially head, neck and central nervous system (CNS) cancers, at that time resulted in statistically imprecise estimates. Here, we leverage the continued follow‐up of the cohort, and an increased number of cancer cases, to further examine the relationship between TETRA use and cancer incidence.

## 2 MATERIALS AND METHODS

### 2.1 Study Sample and Cancer Outcomes

The Airwave Health Monitoring Study is an occupational cohort involving police forces across Great Britain.^11^ Briefly, the study enrolled 53,245 participants between 2004 and 2015, and collected information on socio‐demographic, health, occupational and lifestyle factors via surveys and/or health screenings. Here, we focus on the 49,288 (92.6%) participants with available information on personal radio use.

To define the outcome, we linked participants to national cancer and death registries. We classified as cancer cases any primary malignant neoplasm (MN) except for non‐melanoma skin cancer (ICD10: C44) for which data are inconsistent in the registries. In addition, we included in the cancer case definition: benign neoplasm of cerebral meninges (ICD10: D32.0), neoplasm of unknown behaviour of the supratentorial region of the brain (ICD10: D43.0), benign neoplasm of cranial nerves (ICD10: D33.0), and neoplasm of unknown behaviour of the cranial nerves (ICD10: D43.3). Since TETRA radios are worn on the upper chest with the aerial in proximity to the head and neck when speaking, we separately considered head, neck and CNS cancers based on the National Cancer Institute (NCI) definition of head and neck cancers^12^ to which we added MN of eye and adnexa (ICD10: C69), MN of brain (ICD10: C71), MN of spinal cord and cranial nerves (ICD10: C72), MN of thyroid gland (ICD10: C73) and neoplasms of uncertain and unknown behaviour of the brain (ICD10: D43). Finally, we considered brain and meningeal tumours (ICD10: C71, D32.0 and D33.0), separately from other cancer types. A complete list of cancer cases is available in Table S2.

**FIGURE 1:**
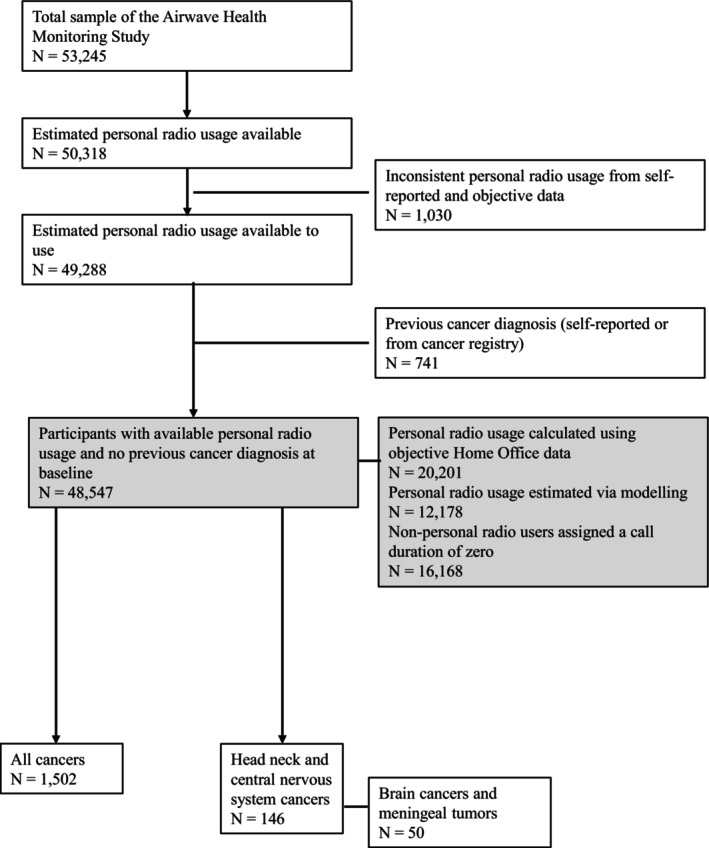
Flowchart of participants in the Airwave Health Monitoring Study included in the analyses.

Participants with a previous cancer diagnosis identified from either cancer registry and/or self‐reported information at recruitment were excluded from the study (Figure 1). For participants with multiple incident cancer diagnoses, we considered the first event only.

### 2.2 Personal radio use, TETRA exposure and personal mobile phone use

Participants provided information on their personal radio and mobile phone use by questionnaire. Information collected included: frequency of TETRA use in press‐to‐talk (PTT)/transmit and/or mobile phone (PSTN) mode (all of the time, some of the time, none of the time), duration (in min) of outgoing calls with TETRA in PTT or PSTN mode over the last shift, total duration (in min) of phone calls made and received on a personal mobile phone in the last 24 hours, and use of hands‐free equipment (yes/no) when talking on a personal mobile phone. In addition, the Home Office provided information on the time and duration of every radio call made and received by participants in the study.

We focus on two main exposures: whether participants were personal radio users, and the estimated average monthly personal radio call duration. The definition of usage is restricted to the time when participants pressed the radio button to speak as using TETRA in listening mode does not result in any RF‐EMF emission. Whenever available (*N* = 20,201), for each participant, we calculated the numbers and duration of calls in the year prior to enrollment in the study from their call records provided by the Home Office. If objective call record data were not available and/or they could not be validated, we assigned an average monthly call duration of zero if participants reported not using a personal radio (*N* = 16,168) or we estimated an average monthly call duration (*N* = 12,178) via an imputation algorithm^13^, ^14^ that included information on self‐reported data on TETRA use, participants' demographics (e.g., age and sex), lifestyle (e.g., physical activity), and occupational (i.e., rank and police force) characteristics.

Detailed information on computation and validation of personal radio call duration, and on the variables used in the imputation algorithm is available in Table S1 and in a previous publication.^13^ Together with the main exposures, for participants with objective data on call records, we computed the average monthly call duration across all years available prior to enrollment (maximum: 10 years, median: 3 years, interquartile range [IQR] = 2–5 years).

### 2.3 Statistical Analysis

We summarised participants' characteristics using median and interquartile range (IQR) for continuous variables and frequency and percentages for categorical variables. Spearman correlation coefficients were calculated to describe the relationship between outcomes, exposures and covariates included in the analysis.

To study the association between personal radio use and cancer incidence, we used Cox proportional hazards models where we assumed that a participant was at risk of developing cancer from the age they entered the study to the age of either their first cancer diagnosis, their death, their withdrawal from the study, or the end of follow‐up (whichever came first). We defined the end of follow‐up based on available dates from the cancer and death registries (1 December 2020). All models were initially adjusted for sex, region of enrollment, education, salary, rank, body mass index (BMI), smoking, number of cigarettes smoked and alcohol use. We checked the proportional hazard assumption by visual inspection of the scaled Schoenfeld residuals. Because the proportional hazard assumption was not satisfied, we stratified each model by sex assuming that men and women have different baseline hazards of developing cancer. We tested for potential non‐linear associations between monthly call duration and cancer risk by modelling the exposure as restricted cubic splines with knots placed at the 33rd and 67th centiles of the observed distribution. Finally, we studied whether using a personal radio without earpiece/microphone modified any association between call duration and cancer risk by adding in the model described above the interaction between average monthly call duration and frequency of personal radio use without earpiece/microphone.

For participants with available self‐reported information on personal mobile phone use, first, we categorised self‐reported call duration in the 24 hours before recruitment into three groups: “never/low” (call duration less than 5 minutes), “medium” (call duration between 5 and 20 minutes) and “high” (call duration greater than 20 minutes) based on tertiles of the observed distribution of call duration. Then, we repeated the main analysis further adjusting for call duration on a mobile phone and use of hands‐free equipment (yes/no) to account for different potential sources of RF‐EMF.

We repeated all analyses restricting the sample to (1) police officers only (i.e., excluding non‐officer staff), (2) participants in forces where at least 5% of objective data could be linked to radio users, and (3) participants who were not personal radio users together with those from whom we had objective data on call duration for at least one year before recruitment in the study.

## 3 RESULTS

Overall, the majority of participants were men (63.5%), personal radio users (66.7%) and police officers (64.4%). Among 48,547 participants who did not have a pre‐baseline cancer diagnosis, a total of 1502 incident cancers were diagnosed during a median (IQR) follow‐up of 11.00 (8.60, 13.36) years (Table 1). Of these diagnoses, 146 were classified as head, neck and CNS cancers (see Table S2 for detailed information on cancer cases). Median (IQR) average monthly call duration in the year prior to enrollment was 8.15 (0.00, 46.60) min (Figure S1). Compared to non‐users, personal radio users were often police officers, younger, and male. Call duration via personal mobile phone was similar in personal radio users and non‐users. A Spearman correlation coefficient of 0.01 between personal radio use and call duration on mobile phone suggested a very weak—if not negligible—relationship between the two variables. Near‐zero correlations were also observed when comparing call duration via mobile phone and call duration via TETRA (Figures S2 and S3).

**TABLE 1:** Characteristics of participants included in the study.

	Median (IQR) or *N* (%)
Age at recruitment (years)	20 (33, 47)
Sex	
Female	17,736 (36.5)
Male	30,811 (63.5%)
Personal radio user	
No	16,168 (33.3%)
Yes	32,370 (66.7%)
Rank	
Officer	31,263 (64.4%)
Staff	11,778 (24.3%)
Missing	5506 (11.5%)
Region	
England	33,846 (70%)
Scotland	6986 (14%)
Wales	7698 (16%)
Missing	17 (0.0%)
Education	
Vocational qualification	2656 (5.5%)
GCSE equivalent or below	12,562 (26%)
A‐levels or equivalence	11,905 (25%)
Bachelor/postgraduate degree	10,360 (21%)
Missing	11,064 (23%)
Salary	
Less than £26,000	8017 (17%)
£26,000–£31,999	6618 (14%)
£32,000–£37,999	8642 (18%)
More than £38,000	13,369 (28%)
Missing	11,901 (25%)
Mobile phone call duration	
Never/low	13,592 (28%)
Medium	19,493 (40%)
High	14,220 (29%)
Missing	1242 (2.6%)
Body mass index (BMI)	
Normal weight	11,631 (24%)
Overweight	17,193 (35%)
Obese	7986 (17%)
Missing	11,193 (35%)
Alcohol drinking	
Past	6180 (13%)
Light and never	20,143 (41%)
Moderate	11,766 (24%)
Heavy	7595 (16%)
Missing	6180 (13%)
Smoking	
Current	5097 (10.5%)
Former	10,594 (22%)
Never	31,981 (66%)
Missing	875 (2.0%)
Daily number of cigarettes	
0–4	1379 (8.8%)
5–9	3090 (20%)
10–15	4250 (27%)
More than 15	6381 (41%)
Missing	591 (3.2%)
Follow‐up time (years)	11.0 (8.6, 13.4)
All cancers	1502 (3.1%)
Head and neck cancers	146 (0.3%)

*Note*: Continuous variables are summarised with median (interquartile range [IQR]); categorical variables are summarised with frequency and percentage. BMI was missing if a participant did not attend the health screening. Other missing values were due to no assessment of those participant's characteristics in a specific version of the questionnaire, or no response received from participants. Alcohol drinking was based on alcohol units calculated from different types of drinks/beverages consumed in the past weeks using sex‐specific cutoffs (light and never: <11 units in men or <7 units in women; moderate: ≤22 units in men and ≤ 15 units in women, heavy: >22 units in men and >15 units in women). Phone call duration was categorised based on tertile (never/low: non users and less than 5 min per day, medium: 5–20 min per day, high: 20 min or more per day). Daily number of cigarettes is reported only for those who are current or former smokers.

**FIGURE 2:**
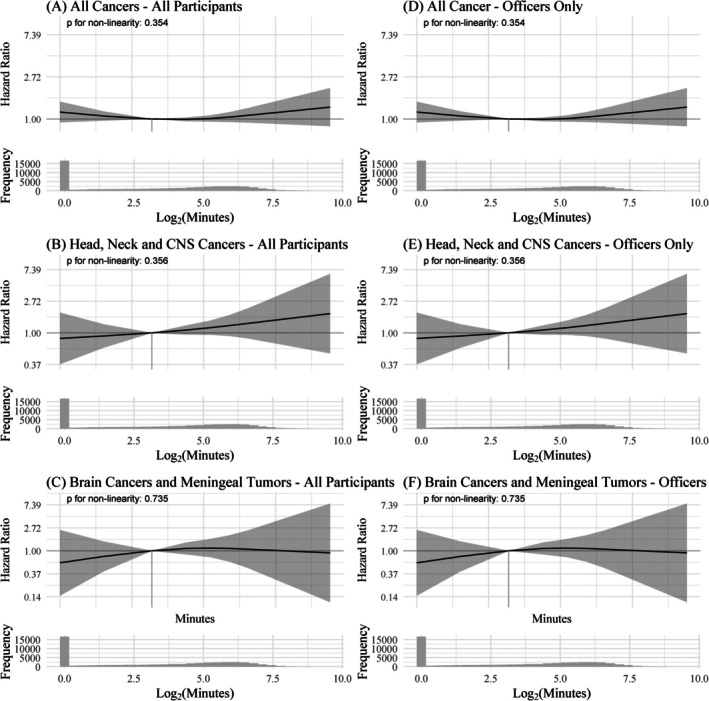
Hazard ratio (HR) and 95% confidence interval (CI) of cancer risk as a function of TETRA call duration. All models were stratified by sex and adjusted for region of enrollment, education, salary, rank, body mass index, smoking status, number of cigarettes smoked per day, and alcohol use. The logarithm of call duration using TETRA was modelled using restricted cubic splines with three knots. P in the plot refers to the *p*‐value testing non‐linearity. Panels (A), (B) and (C) summarise results from analyses including all participants and all cancers, head neck and CNS cancers, and brain cancers and meningeal tumours respectively. Panels (D), (E) and (F) summarise results from analyses including only police officers and all cancers, head neck and CNS cancers, and brain cancers and meningeal tumours respectively.

There was no association between personal radio use and the risk of developing cancer. Compared to participants who did not use a personal radio, users had 4% (HR: 0.96, 95% CI: 0.80, 1.16), 26% (HR: 0.74, 95% CI: 0.39, 1.38) and 17% (HR: 0.83, 95% CI: 0.29, 2.40) lower hazard of developing all cancers, head, neck and CNS cancers, and brain cancer and meningeal tumours respectively. Overall, monthly call duration in the year before recruitment was not associated with cancer risk; however, results in Table 2 suggest a potential—although not statistically significant—9% higher risk of developing head, neck and CNS cancers (HR: 1.09, 95% CI: 0.97, 1.22) when doubling minutes of monthly call duration. There was no evidence of a non‐linear association between call duration and cancer risk (Figure 2 and Table S3). Compared to participants with an average of 8.15 minutes of monthly call duration (equivalent to the observed median call duration), those in the 90th percentile (averaging 92.8 minutes per month) had a 9% higher risk of developing all cancers (HR: 1.09, 95% CI: 0.92, 1.51), a 40% (HR: 1.40, 95% CI: 0.85, 2.23) higher risk of developing head, neck and CNS cancers and a 4% (HR: 1.04, 95% CI: 0.45, 2.39) higher risk of developing brain cancers and meningeal tumours. In contrast, participants who did not use a personal radio (i.e., those in the 25th percentile or lower with zero minutes of call duration) had an 18% (HR: 1.18, 95% CI: 0.82, 1.51) higher risk of all cancers, a 15% (HR: 0.85, 95% CI: 0.38, 1.92) lower risk of head, neck and CNS cancers, and a 41% (HR: 0.59, 95% CI: 0.14, 2.45) lower risk of brain cancers and meningeal tumours. There was no evidence that the frequency of personal radio use without an earpiece/microphone modified the association between call duration and risk of cancer (Table S4). Results were similar when we restricted our sample only to police officers who were cancer‐free at recruitment (*N* = 31,263). There were no discernible associations between mobile phone use and cancer risk (Table S5).

**TABLE 2:** Hazard ratio (HR) and 95% confidence interval (CI) estimating the association between cancer, personal radio use and call duration.

	All cancer		Head, neck and CNS cancer		Brain cancer and meningeal tumours	
	*N* cases	HR (95% CI)	*N* cases	HR (95% CI)	*N* cases	HR (95% CI)
Participants (offices + staff) cancer‐free at baseline (*N* = 48,547)						
Personal radio use						
No	698	Reference	54	Reference	16	Reference
Yes	804	0.96 (0.80, 1.16)	92	0.74 (0.39, 1.38)	34	0.83 (0.29, 2.40)
Doubling of minutes of use		1.00 (0.96, 1.04)		1.09 (0.97, 1.22)		1.06 (0.88, 1.27)
Officers cancer‐free at baseline (*N* = 31,263)						
Personal radio use						
No	186	Reference	17	Reference	5	Reference
Yes	571	0.82 (0.64, 1.04)	68	0.80 (0.38, 1.71)	25	0.91 (0.24, 3.48)
Doubling of minutes of use		1.01 (0.97, 1.05)		1.03 (0.91, 1.17)		1.02 (0.83, 1.26)

*Note*: All models were stratified by sex and adjusted for region of enrollment, education, salary, rank, body mass index, smoking, number of cigarettes smoked and alcohol use, CNS, central nervous system.

Sensitivity analyses including only forces where at least 5% of the personal radio use could be linked to objective data (*N* = 38,908), led to similar conclusions (Table 3). We found that officers who used the radio had a 27% (HR: 0.73, 95% CI: 0.56, 0.96) lower risk of developing all cancer compared to those who did not use the radio. This might be explained by selection bias: officers who used the radio were younger, had lower BMI and were less likely to be heavy drinkers and/or smokers (Table S6). Finally, when restricting the analysis to participants that were not personal radio users and those with available objective data on personal radio use for one or more years before recruitment in the study (*N* = 36,369), we found no associations between personal radio use or duration of use and cancer incidence regardless of whether we considered average usage in the year before recruitment or across all years prior to enrollment (Tables S7 and S8).

**TABLE 3:** Hazard ratio (HR) and 95% confidence interval (CI) estimating the association between cancer, personal radio use and call duration for forces with more than 5% of objective data among personal radio users.

	All cancer		Head, neck and CNS cancer		Brain cancer and meningeal tumours	
	*N* cases	HR (95% CI)	*N* cases	HR (95% CI)	*N* cases	HR (95% CI)
Participants (officer + staff) cancer‐free at baseline (*N* = 38,908)						
Personal radio use						
No	603	Reference	45	Reference	14	Reference
Yes	700	0.93 (0.76, 1.13)	83	0.79 (0.40, 1.55)	31	0.88 (0.29, 2.69)
Doubling of minutes of use		1.01 (0.97, 1.04)		1.09 (0.97, 1.23)		1.06 (0.88, 1.29)
Officers cancer‐free at baseline (*N* = 24,406)						
Personal radio use						
No	100	Reference	13	Reference	4	Reference
Yes	497	0.73 (0.56, 0.96)	61	0.77 (0.33, 1.76)	22	0.83 (0.19, 3.55)
Doubling of minutes of use		1.02 (0.98, 1.07)		1.05 (0.92, 1.19)		1.04 (0.84, 1.30)

*Note*: All models were stratified by sex and adjusted for region of enrollment, education, salary, rank, body mass index (BMI), smoking, number of cigarettes smoked and alcohol use. CNS, central nervous system.

## 4 DISCUSSION

Overall, there was no evidence for an association between TETRA use, average call duration in the year before recruitment and/or across all years before recruitment and risk of cancer. Results are similar to those previously published in Airwave,^1^ but the increased number of newly developed cancer cases led to more precise estimates and narrower confidence intervals. Other epidemiological studies on occupational RF‐EMF exposure likewise did not find any association with the risk of developing cancer.^15^, ^16^

More generally, studies on the effect of RF‐EMF exposure on health outcomes provide conflicting results: small effects of TETRA signal on the electroencephalogram were consistent with vagal nerve stimulation in the chest in one study,^4^ but other studies did not find any effect when looking at head exposure and cognitive function.^5^, ^6^ The INTERPHONE study, a case–control study across 13 countries, reported an excess of glioma and meningioma in the top decile of cumulative use of mobile phones,^17^ while data from the Million Women Survey and the Cohort Study on Mobile Phones and Health (COSMOS),^2^, ^18^ two large prospective cohort studies, showed no evidence of an association between self‐reported mobile phone use and cancer risk. In the present analysis, we also found no association between self‐reported call duration on a mobile phone, all cancers, head, neck and CNS cancers risk and/or brain cancer and meningeal tumours. Similar to previous studies,^1^, ^2^, ^18^ we used call duration as a proxy of RF‐EMF exposure instead of SAR as the latter depends on factors such as device type, position and distance from the body that could not be reliably estimated.

The size of the cohort and the availability of survey data, and of objectively measured use of TETRA are strengths of our study. To include a higher number of participants in the analyses, we used as primary exposure one that combined objective data—for participants with available linkage to the Home Office call records—with model estimated data—for participants without available linkage. In a previous paper,^13^ we showed a high correlation between objective and model‐estimated call duration with the latter slightly underestimating duration for the highest users. Moreover, when limiting the analysis to participants who either did not use the radio or had objective information on call duration, we observed similar results to those of our primary exposure suggesting that the impact of using call duration estimated via modelling for a portion of the participants was minimal. Finally, compared with our previous analysis, we considered mobile phone use as well as frequency of personal radio use without an earphone/microphone. However, there are limitations. First, while the number of cancer cases has doubled compared to our previous analysis, the number of head, neck and CNS cancers and brain cancers and meningeal tumours, is still relatively low, limiting the ability to detect any increase in risk, and hindering clear understanding of the results. For instance, while we observed a 26% higher risk of developing head, neck and CNS cancers in participants who are self‐reported personal radio users, the CI associated with the estimated risk suggests anything between a 61% lower to a 38% higher risk of head, neck and CNS cancers. Second, we were not able to link to the objective usage records for many officers including those in the Metropolitan police (i.e., the largest force enrolled in the study with approximately 8000 participants). Each police force had its own rules for linking objective data to participants. To prevent potential linkage errors and minimise any source of bias, we did not attempt linkage for participants in forces that either (1) did not provide radio usage at the time of recruitment or (2) provided linkage rules that could not uniquely identify a person. Moreover, for participants in forces with clear linkage rules, we only considered a successful linkage that passed our validation steps outlined in Appendix S1. Although this reduced the number of participants with direct linkage, it allowed us to include the most accurate measure of TETRA use. Further, for some users, we were only able to include a few months of usage data, which could potentially lead to an over‐ or under‐estimation of TETRA call duration if the available months were not representative of a participant's typical usage. However, results from the sub‐group analysis that only included participants with at least one year of objective data were similar to those from the overall analysis, suggesting that the duration of available TETRA usage data did not unduly affect the results. Information on the location of TETRA was available only for a small subset of participants recruited at the beginning of the study (*N* = 3407). Due to limited numbers of new cancer cases (96 total cancers with 11 head, neck and CNS cancers) we could not study whether the location of the radio modified the association between call duration and cancer. Finally, self‐reported data on mobile phone use might not be fully representative of overall use as they refer to the 24 hours before recruitment in the study, and they can be influenced by systematic or random measurement errors.^19^, ^20^

In conclusion, we found no evidence of an association between TETRA use and risk of cancer. Whilst the results from the linear and non‐linear models cannot exclude a modestly greater risk with higher TETRA usage, the wide confidence intervals are consistent with both substantially increased and substantially decreased risks, and thus a definite conclusion cannot be reached. Longer follow‐up time would give us the opportunity to accrue more cancer cases, increase precision of estimated risks, ease the interpretation of the results, and analyse whether specific groups of participants have higher risk of developing cancer.

## AUTHOR CONTRIBUTIONS

**Chiara Di Gravio:** Formal analysis; writing – review and editing; writing – original draft; methodology; visualization. **Paul Elliott:** Conceptualization; supervision; resources; writing – review and editing. **David C. Muller:** Supervision; conceptualization; writing – review and editing; methodology.

## FUNDING INFORMATION

The first phase of the Study was funded by the Home Office (grant number 780‐TETRA) and the Economic and Social Research Council and Medical Research Council (MR/R023484/1) with additional support from the National Institute for Health Research (NIHR) Health Protection Research Unit in Chemical and Radiation Threats and Hazards (NIHR‐200922). The current phase is partially supported by the NIHR HPRU in Radiation Threats and Hazards (NIHR‐207424), a partnership between the UK Health Security Agency and Imperial College London.

## CONFLICT OF INTEREST STATEMENT

The authors declare no conflict of interest.

## ETHICS STATEMENT

The Airwave Health Research Tissue Bank has ethical approval through the North West Haydock Research Ethics Committee (REC23/NW/0350; IRAS 333243). Each participant provided informed written consent. The study was performed in accordance with the Declaration of Helsinki.

## Supporting information


**APPENDIX S1:** Supporting information.

## Data Availability

The datasets generated and analysed during the current study are not publicly available due to restrictions imposed by the data provided (NHS) but are available from the corresponding author on reasonable request.
