# Supplementary material for: Personal radio use and risk of cancers among police officers in Great Britain: Results from the airwave health monitoring study
Source: Int J Cancer. 2025 Nov 18;158(9):2289–97. doi: 10.1002/ijc.70255 (PMC12963709; doi:10.1002/ijc.70255)
Supplement: Supplementary file 1 — APPENDIX S1: Supporting information. [file IJC-158-2289-s001.pdf]

Supplementary Material for

PERSONAL RADIO USE AND RISK OF CANCERS AMONG POLICE OFFICERS IN GREAT BRITAIN: RESULTS FROM THE AIRWAVE HEALTH MONITORING STUDY

Chiara Di Gravio, Paul Elliott, David C Muller

Table of contents:

Supplementary Methods

Supplementary Tables 1 – 8

Supplementary Figures 1 -3

Supplementary Methods

Overview of the Algorithm for the Computation of TETRA Call Duration

The algorithm for computing call duration consists of three distinct steps: first we linked data from the Home Office to each participant, then we checked whether the linkage is valid; finally, for participants with no available linkage we predict their call duration via imputation. The three steps are briefly summarized below. Additional information can be found in previously published papers (1,2).

Step 1. Linking TETRA Call Duration

Data on TETRA use provided by the Home Office consist of one observation per radio transmission. TETRA call duration linkage was based on 1) the individual short subscriber identifying (ISSI) number, 2) the police employee's collar number (i.e., a unique identification number for each member of the police and/or staff), and 3) the police force that employed each participants. In the Home Office data, records (i.e., calls) are identified by the ISSI number, the date and time of the call, and the number of base. Whenever available, using force-specific rule, we linked each ISSI number to individual police employee's collar number. Among all police forces, four were excluded from the analysis: two provided non-unique ISSI rules (i.e., two ISSI numbers could be derived from the same collar number), one did not have any call record at time of enrolment in the study, and one had a poorer linkage performance.

Step 2. Validating the Linkage

To validate the linkage, we compared the linked data from the Home Office data with four data sources:

1) self-reported TETRA use (yes/no): self-reported and operator data were labelled "Consistent" only when some operator-derived records were found in the year prior to recruitment. Data were labelled as "Consistent" for 94% of the participants.

2) self-reported information on time and date of last shift: self-reported and operator data were labelled "Consistent" if a participant reported any minutes or calls during the shift, and we found some operator-derived records on the shift date (allowing an extra hour before the start and after the end of the shift to account for potential misreporting of shift time). Data were labelled as "Consistent" for 57% of the participants.

3) self-reported 7-day radio usage diary: self-reported and operator data were labelled "Consistent" when the participants reported some minutes or some calls in their usage diary, and some operator-derived records were found on that day, and/or when no operator-derived records were found in days when a participant did not report usage. Data were labelled as "Consistent" for 80% of the participants.

4) sickness absences: self-reported and operator data were labelled "Consistent" there was no objective record during participants' sickness absences. Data were labelled as "Consistent" for 28% of the participants.

Linkage between operator data and self-reported data was considered successful if at least two of the four sources listed above were labelled as "Consistent".

Step 3. Computation of TETRA Call Duration

For participants with a successful linkage, we used call duration computed using network-operator data provided by the Home Office. When data from the Home Office were not available, or the linkage was unsuccessful:

1) if a participant reported not using TETRA, then we assigned call duration to zero,
2) if a participant reported using TETRA, we predicted their call duration from their socio-demographics characteristics and self-reported information on TETRA use, as well as characteristics of participants for whom we had TETRA use from the Home Office (see full list of variables in Supplementary Table 1). Prediction was done using a gradient boosting method, and method's performance was validated on a test dataset.

Supplementary Tables

Supplementary Table 1. Variables included in the gradient boosting algorithm to estimate TETRA usage for participants who had no successful linkage to Home Office Data and were personal radio users (all variables were self-reported by participants at time of recruitment.

**Socio-demographic variables**	Sex, age, education, ethnicity, salary, satisfaction with standard of living, marital status, number of people in the household
**Occupational factors**	Rank, number of years in the current role, years of service, current role, current police force, work environment (having to work hard, having an excessive amount of work, having a high skill level, having a lot to say about what happens at work on a normal day, having freedom to decide how to work, having the chance to be creative, having colleagues and superiors support, job-satisfaction, part-time worker, overtime worker, night-shift
**Physical health status**	Hypertension, body mass index, fat percentage, abdominal obesity, high cholesterol, diabetes, abnormal electrocardiogram, chronic disease, number of sickness leave day in the past year, number of visits to the GP in the past year
**Mental health status**	Depression (PHQ-9 score), anxiety, post-traumatic stress disorder (PTSD)
**Lifestyle factors**	Hours of sleep, smoking status, drinking status, consumption of red meat, consumption of fruit

	and vegetables, hours of physical activity, hours of spent sitting in a week, mobile phone use (yes/no)
**Personal radio usage**	Usual usage is reported in the questionnaire, participants still uses an old analogue radio, number of years since starting using TETRA, type of last shift (early morning, morning, evening, night), duration of last shift, number of daily calls in the last shift, total duration of calls in the last shift.
	Report of headache, warming sensation of face or ear, other symptoms after using a personal radio and/or a personal mobile phone
**Usage of other types of radio**	Usage of desk-mounted radio, motorbike-mounted radio, covert radio, car-mounted radio, pool radio (number of calls and duration).
**Seasonality**	Year, month and day in which the questionnaire was filled

Supplementary Table 2. Detailed information on cancers included in the analysis. MN, malignant neoplasm. CNS, central nervous system.

ICD10 code	Description	N (%)	Head, neck and CNS cancers
C01	MN of base of tongue	11 (0.73)	√
C02	MN of other and unspecified parts of tongue	7 (0.47)	√
C03	MN of gum	1 (0.07)	√
C06	MN of other and unspecified parts of mouth	2 (0.13)	√
C07	MN of parotid gland	3 (0.20)	√
C09	MN of tonsil	20 (1.33)	√
C10	MN of oropharynx	3 (0.20)	√
C11	MN of nasopharynx	5 (0.33)	√
C13	MN of hypopharynx	1 (0.07)	√
C15	MN of oesophagus	17 (1.13)	
C16	MN of stomach	18 (1.20)	
C17	MN of small intestine	4 (0.26)	
C18	MN of colon	84 (5.59)	
C19	MN of rectosigmoid junction	9 (0.60)	
C20	MN of rectum	53 (3.52)	
C21	MN of anus and anal canal	5 (0.33)	
C22	MN of liver and intrahepatic bile ducts	6 (0.39)	
C24	MN of other and unspecified parts of biliary tract	6 (0.39)	
C25	MN of pancreas	26 (1.73)	
C26	MN of others and ill-defined digestive organ	2 (0.13)	
C30	MN of nasal cavity and middle ear	1 (0.07)	√
C31	MN of accessory sinuses	1 (0.07)	√
C32	MN of larynx	2 (0.13)	√
C34	MN of bronchus and lung	44 (2.93)	
C37	MN of thymus	1 (0.07)	
C38	MN of heart, mediastinum, and pleura	3 (0.20)	
C41	MN of bone and articular cartilage of other and unspecified sites	1 (0.07)	
C43	Malignant melanoma of skin	155 (10.3)	
C45	Mesothelioma	3 (0.20)	
C48	MN of retro peritoneum and peritoneum	2 (0.13)	
C49	MN of other connective and soft tissue	13 (0.87)	
C50	MN of breast	286 (19.0)	

C51	MN of vulva	4 (0.26)	
C53	MN of cervix uteri	1 (0.07)	
C54	MN of corpus uteri	26 (1.73)	
C56	MN of ovary	24 (1.60)	
C57	MN of other and unspecified female genital organs	1 (0.07)	
C60	MN of penis	3 (0.20)	
C61	MN of prostate	274 (18.2)	
C62	MN of testis	32 (2.13)	
C63	MN of other and unspecified male genital organs	2 (0.13)	
C64	MN of kidney, except renal pelvis	58 (3.86)	
C65	MN of renal pelvis	2 (0.13)	
C67	MN of bladder	19 (1.26)	
C69	MN of eye and adnexa	4 (0.26)	√
C71	MN of brain	50 (3.33)	√
C72	MN of spinal cord, cranial nerves, and other	2 (0.13)	√
C73	MN of thyroid gland	32 (2.13)	√
C74	MN of adrenal gland	2 (0.13)	
C75	MN of other endocrine glands and related structure	1 (0.07)	
C80	MN without specification of site	12 (0.80)	
C81	Hodgkin's disease	14 (0.93)	
C82	Follicular [nodular] non-Hodgkin's lymphoma	16 (1.07)	
C83	Diffuse non-Hodgkin's lymphoma	30 (1.99)	
C84	Peripheral and cutaneous T-cell lymphomas	5 (0.33)	
C85	Other and unspecified types of non-Hodgkin's lymphoma	11 (0.73)	
C88	Malignant immunoproliferative diseases	1 (0.07)	
C90	Multiple myeloma and malignant plasma cell neoplasms	12 (0.80)	
C91	Lymphoid leukaemia	22 (1.46)	
C92	Myeloid leukaemia	25 (1.66)	
C93	Monocytic leukaemia	1 (0.07)	
D43	Neoplasm of uncertain or unknown behaviour of brain	1 (0.07)	√
**Total**		**1,502**	**146**

Supplementary Table 3. Hazard ratio and 95% confidence interval from Cox models with restricted cubic splines shown for different centiles relative to the median (8.15 minutes per month when including all participants and 22.7 minutes per month for officers only). Models were stratified by sex and adjusted for region of enrollment, education, salary, rank, body mass index, smoking, number of cigarettes smoked and alcohol use. CNS, central nervous system.

		All Cancer	Head, Neck and CNS Cancer	Brain Cancer and Meningeal Tumors
		HR (95% CI)	HR (95% CI)	HR (95% CI)
**Participants (offices + staff) cancer-free at baseline (N = 48,547)**				
Percentile	Call Duration per Month (min)			
10%	0	1.18 (0.92, 1.51)	0.85 (0.38, 1.92)	0.59 (0.14, 2.45)
25%	0	1.18 (0.94, 1.51)	0.85 (0.38, 1.92)	0.59 (0.14, 2.45)
50%	8.15	Reference	Reference	Reference
75%	46.6	1.03 (0.94, 1.13)	1.23 (0.94, 1.62)	1.10 (0.68, 1.77)
90%	92.8	1.09 (0.92, 1.30)	1.38 (0.85, 2.23)	1.04 (0.45, 2.39)
**Officers cancer-free at baseline (N = 31,263)**				
Percentile	Call Duration per Month (min)			
10%	0	1.13 (0.81, 1.57)	1.12 (0.41, 3.05)	0.72 (0.12, 4.40)
25%	1.96	1.04 (0.88, 1.24)	1.02 (0.61, 1.73)	0.84 (0.32, 2.20)
50%	22.7	Reference	Reference	Reference
75%	64.2	1.08 (0.96, 1.21)	1.14 (0.83, 1.56)	0.96 (0.56, 1.54)
90%	109.2	1.14 (0.94, 1.40)	1.25 (0.72, 2.19)	0.92 (0.36, 2.36)

Supplementary Table 4. Estimated hazard ratio (HR) and 95% confidence interval (CI) estimating the association between doubling minutes of TETRA use and risk of developing cancer according to frequency of using a personal radio without an earpiece/microphone. Models were stratified by sex and adjusted for region of enrollment, education, salary, rank, body mass index, smoking, number of cigarettes smoked and alcohol use.

	All Cancer		Head, Neck and CNS Cancers	
	N cases	HR (95% CI)	N cases	HR (95% CI)
**Participants (officers + staff) cancer-free at baseline (N = 48,547)**				
**Frequency of using a personal radio without an earpiece/microphone**				
None of the time	756	0.99 (0.04, 1.04)	58	0.99 (0.82, 1.18)
Some of the time	311	0.99 (0.94, 1.04)	34	1.08 (0.92, 1.28)
All of the time	322	1.04 (0.98, 1.10)	43	1.06 (0.89, 1.26)
**Officers cancer-free at baseline (N = 31,263)**				
**Frequency of using a personal radio without an earpiece/microphone**				
None of the time	222	0.97 (0.90, 1.03)	19	0.93 (0.73, 1.18)
Some of the time	238	0.99 (0.94, 1.06)	26	1.01 (0.84, 1.21)
All of the time	274	1.03 (0.96, 1.10)	38	1.03 (0.86, 1.24)

Due to small numbers of brain cancer and meningeal tumors in each call, we could not estimate hazard rations and 95% confidence intervals.

Supplementary Table 5. Hazard ratio (HR) and 95% confidence interval (CI) estimating the association between cancer, personal radio use and call duration from TETRA and mobile phone. Models were stratified by sex and adjusted for region of enrollment, education, salary, rank, body mass index, smoking, number of cigarettes smoked, alcohol use and use of hands-free device when talking on a mobile phone. CNS, central nervous system.

	All Cancer		Head, Neck and CNS Cancer		Brain Cancer and Meningeal Tumors	
	N cases	HR (95% CI)	N cases	HR (95% CI)	N cases	HR (95% CI)
**Participants (officers + staff) cancer-free at baseline (N = 48,547)**						
Personal radio use						
No	698	Reference	54	Reference	16	Reference
Yes	804	0.95 (0.79, 1.15)	91	0.73 (0.39, 1.38)	34	0.82 (0.28, 2.34)
Doubling of minutes of TETRA use		1.00 (0.97, 1.04)		1.09 (0.97, 1.22)		1.06 (0.88, 1.28)
Self-reported mobile phone call duration						
Never/Low	488	1.08 (0.95, 1.22)	45	1.08 (0.72, 1.60)	14	0.94 (0.48, 1.85)
Medium	587	Reference	58	Reference	24	Reference
High	370	1.06 (0.92, 1.21)	38	1.08 (0.71, 1.63)	10	0.67 (0.32, 1.42)
**Officers cancer-free at baseline (N = 31,263)**						
Personal radio use						
No	186	Reference	17	Reference	5	Reference
Yes	571	0.81 (0.64, 1.03)	68	0.82 (0.38, 1.74)	25	0.99 (0.26, 3.80)
Doubling of minutes of TETRA use		1.01 (0.97, 1.06)		1.03 (0.91, 1.17)		1.00 (0.81, 1.24)
Self-reported mobile						

phone call duration						
Never/Low	211	0.94 (0.78, 1.12)	24	0.96 (0.57, 1.63)	9	1.03 (0.44, 2.40)
Medium	306	Reference	35	Reference	14	Reference
High	225	1.09 (0.92, 1.31)	23	1.02 (0.59, 1.74)	5	0.55 (0.19, 1.55)

Phone call duration was categorized based approximately on thirds (Never/low: non-users and less than 5 minutes per day, medium: 5 to 20 minutes per day, high: 20 minutes or more per day). Participants with missing phone call duration are not included in the table.

Supplementary Table 6. Characteristics of police officers by personal radio use after excluding forces with less than 5% of objective data available (N = 24,406).

	All	User	Non-user
	N = 24,406	N = 21,352	N = 3,054
**Age, median (IQR)**	40 (33, 45)	39 (33, 45)	45 (40, 49)
**Sex, n (%)**			
Female	6,335 (26)	5,325 (25)	1,010 (33)
Male	18,071 (74)	16,027 (75)	2,044 (67)
**Region, n (%)**			
England	15,138 (62)	13,034 (61)	2,104 (69)
Scotland	5,987 (25)	5383 (25)	604 (20)
Wales	3, 281 (13)	2935 (14)	346 (11)
**Education, n (%)**			
Vocational qualifications	1,333 (6)	1,200 (6)	133 (4)
GCSE equivalent or below	6,703 (27)	5,740 (27)	963 (32)
A-levels or equivalent	6,966 (29)	6,151 (29)	815 (27)
Bachelor / Postgraduate	547 (22)	4,802 (22)	668 (22)
Missing	3,934 (16)	3,459 (16)	475 (16)
**Salary, n (%)**			
less than £26,000	1,938 (8)	1,729 (8)	209 (7)
£26,000 - £31,999	4,341 (18)	4,098 (19)	243 (8)
£32,000 - £37,999	6,471 (27)	5,686 (27)	785 (26)
More than £38000	7,208 (30)	5,932 (28)	1276 (42)
Missing	3943 (16)	3,907 (18)	541 (18)
**Body mass index, n (%)**			
Normal weight	5,638 (23)	5,037 (24)	601 (20)
Overweight	10,054 (41)	8,817 (41)	1,237 (41)
Obese	4,355 (18)	3,671 (17)	684 (22)
Missing	4,359 (18)	3,827 (18)	532 (17)
**Alcohol drinking, n (%)**			
Past	1,369 (6)	1,192 (6)	177 (6)
Light and never	10,594 (43)	9,495 (44)	1,099 (36)
Moderate	6,656 (27)	5,807 (27)	849 (28)
Heavy	4,437 (18)	3,700 (17)	737 (24)
Missing	1,350 (6)	1,158 (5)	192 (6)
**Smoking, n (%)**			
Never	16,782 (69)	14,801 (69)	1,981 (65)
Former	5,041 (21)	4,927 (20)	20 (1)
Current	2,325 (9)	2,016 (10)	744 (24)
Missing	258 (1)	238 (1)	309 (10)
**Daily number of cigarettes, n (%)**			
0-4	17,364 (71)	15,310 (72)	2,054 (67)

5-9	(1,468 (6))	1,317 (6)	151 (5)
10-15	2,026 (8)	1,736 (8)	290 (10)
More than 15	3,010 (12)	2,508 (12)	502 (16)
Missing	538 (2)	481 (2)	57 (2)

Supplementary Table 7. Hazard ratio (HR) and 95% confidence interval (CI) estimating the association between cancer, personal radio use and call duration from TETRA for participants with valid linkage to objective data. Models were stratified by sex and adjusted for region of enrollment, education, salary, rank, body mass index, smoking, number of cigarettes smoked and alcohol use. CNS, central nervous system.

	All Cancer		Head, Neck and CNS Cancer		Brain Cancer and Meningeal Tumors	
	N cases	HR (95% CI)	N cases	HR (95% CI)	N cases	HR (95% CI)
**Participants (offices + staff) cancer-free at baseline (N = 36,369)**						
Personal radio use						
No	521	Reference	54	Reference	5	Reference
Yes	698	0.97 (0.77, 1.22)	67	0.92 (0.43, 1.96)	18	1.36 (0.42, 4.43)
Doubling of minutes of TETRA use		1.00 (0.96, 1.05)		1.08 (0.95, 1.22)		1.03 (0.84, 1.26)
**Officers cancer-free at baseline (N = 21,866)**						
Personal radio use						
No	186	Reference	17	Reference	5	Reference
Yes	382	0.78 (0.58, 1.04)	49	0.89 (0.37, 2.15)	18	1.18 (0.26, 5.40)
Doubling of minutes of TETRA use		1.02 (0.97, 1.07)		1.03 (0.89, 1.19)		1.00 (0.79, 1.28)

Supplementary Table 8. Hazard ratio (HR) and 95% confidence interval (CI) estimating the association between cancer, personal radio use and call duration from TETRA for participants with at least one year of recorded call duration. Models were stratified by sex and adjusted for region of enrollment, education, salary, rank, body mass index, smoking, number of cigarettes smoked and alcohol use. CNS, central nervous system.

	All Cancer		Head, Neck and CNS Cancer		Brain Cancer and Meningeal Tumors	
	N cases	HR (95% CI)	N cases	HR (95% CI)	N cases	HR (95% CI)
**Participants (offices + staff) cancer-free at baseline (N = 36,369)**						
Personal radio use						
No	698	Reference	54	Reference	16	Reference
Yes	521	1.02 (0.79, 1.32)	67	1.23 (0.56, 2.67)	26	2.19 (0.69, 7.13)
Doubling of minutes of TETRA use		0.99 (0.95, 1.04)		1.01 (0.88, 1.16)		0.92 (0.74, 1.13)
**Officers cancer-free at baseline (N = 21,866)**						
Personal radio use						
No	186	Reference	17	Reference	5	Reference
Yes	382	0.81 (0.59, 1.12)	49	1.15 (0.46, 2.92)	18	1.73 (0.38, 7.70)
Doubling of minutes of TETRA use		1.01 (0.95, 1.07)		0.97 (0.83, 1.39)		0.92 (0.71, 1.19)

Supplementary Figure 1. Distribution of average monthly call duration for all participants in the Airwave Study and for officers only.

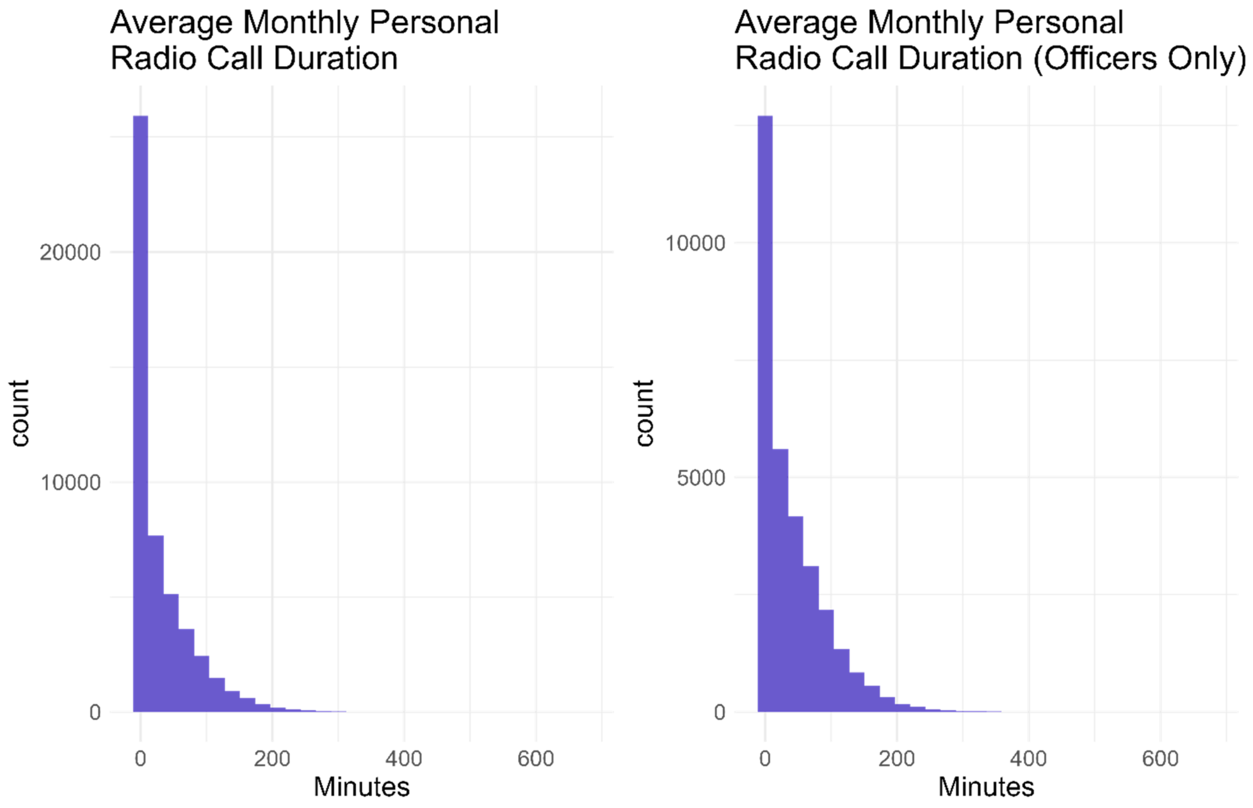

Supplementary Figure 2. Distribution of personal mobile phone call duration according to whether participants were personal radio use. Call duration on mobile phone refer to the 24 hours before recruitment.

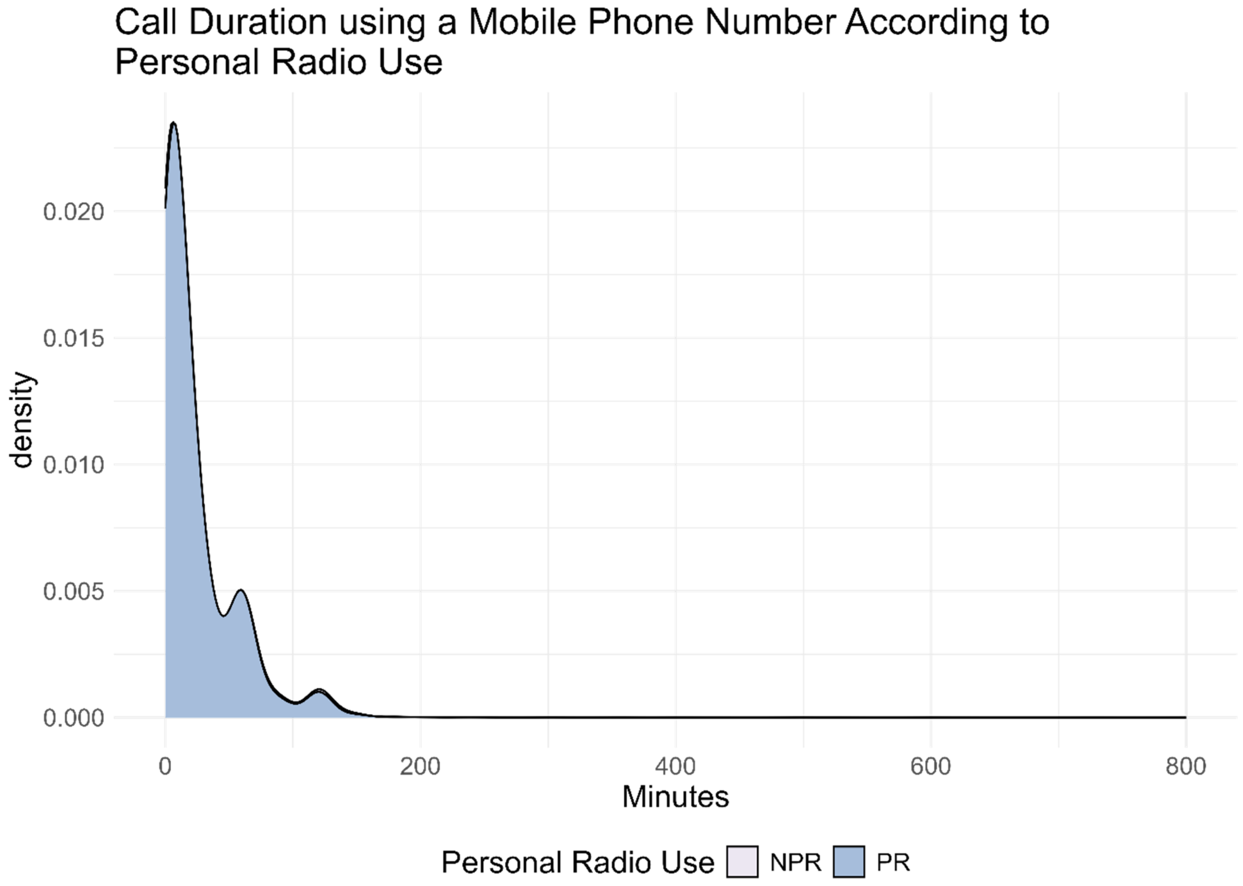

Supplementary Figure 3. Spearman's correlation for the exposures and covariates included in the primary analysis. CD, call duration, cigarettes, number of cigarettes per day. Because salary, and number of cigarettes per day were categorical variables, for both variables, the mid-point of each category was used to compute the correlation coefficient.

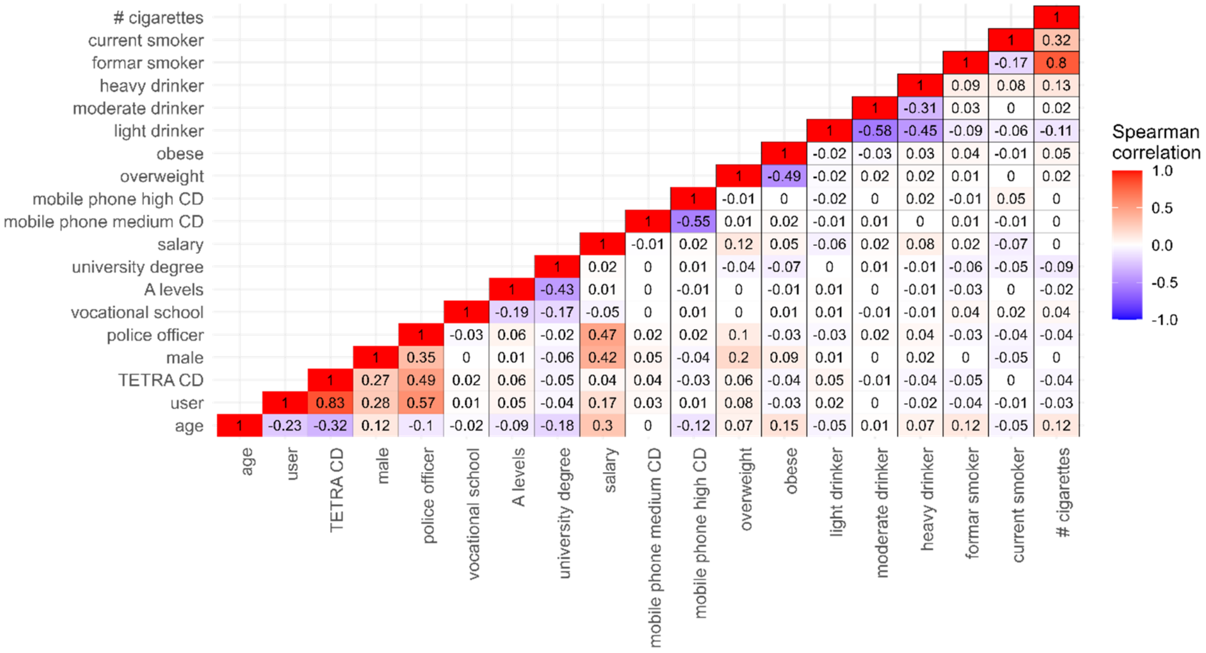

Supplementary Reference

1. Vergnaud AC, Aresu M, McRobie D, Singh D, Spear J, Heard A, et al. Validation of objective records and misreporting of personal radio use in a cohort of British Police forces (the Airwave Health Monitoring Study). Environ Res. 2016 Jul 1;148:367–75.

2. Vergnaud AC, Aresu M, Kongsgård HW, McRobie D, Singh D, Spear J, et al. Estimation of TETRA radio use in the Airwave Health Monitoring Study of the British police forces. Environ Res. 2018 Nov 1;167:169–74.
